# Functional properties of GABA_A_ receptors of AII amacrine cells of the rat retina

**DOI:** 10.3389/fopht.2023.1134765

**Published:** 2023-04-05

**Authors:** Pablo Beltrán-Matas, Espen Hartveit, Margaret L. Veruki

**Affiliations:** Department of Biomedicine, University of Bergen, Bergen, Norway

**Keywords:** retina, AII amacrine cell, GABAA receptors, GABAA alpha3 subunit, GABAA alpha2 subunit, rod pathway

## Abstract

Amacrine cells are a highly diverse group of inhibitory retinal interneurons that sculpt the responses of bipolar cells, ganglion cells, and other amacrine cells. They integrate excitatory inputs from bipolar cells and inhibitory inputs from other amacrine cells, but for most amacrine cells, little is known about the specificity and functional properties of their inhibitory inputs. Here, we have investigated GABA_A_ receptors of the AII amacrine, a critical neuron in the rod pathway microcircuit, using patch-clamp recording in rat retinal slices. Puffer application of GABA evoked robust responses, but, surprisingly, spontaneous GABA_A_ receptor-mediated postsynaptic currents were not observed, neither under control conditions nor following application of high-K^+^ solution to facilitate release. To investigate the biophysical and pharmacological properties of GABA_A_ receptors in AIIs, we therefore used nucleated patches and a fast application system. Both brief and long pulses of GABA (3 mM) evoked GABA_A_ receptor-mediated currents with slow, multi-exponential decay kinetics. The average weighted time constant (τ_w_) of deactivation was ~163 ms. Desensitization was even slower, with τ_w_ ~330 ms. Non-stationary noise analysis of patch responses and directly observed channel gating yielded a single-channel conductance of ~23 pS. Pharmacological investigation suggested the presence of α2 and/or α3 subunits, as well as the γ2 subunit. Such subunit combinations are typical of GABA_A_ receptors with slow kinetics. If synaptic GABA_A_ receptors of AII amacrines have similar functional properties, the slow deactivation and desensitization kinetics will facilitate temporal summation of GABAergic inputs, allowing effective summation and synaptic integration to occur even for relatively low frequencies of inhibitory inputs.

## Introduction

Inhibitory interneurons of the inner retina play a critical role in neural computations that establish parallel channels of visual information (reviewed in (1)). These neurons, called amacrine cells, make inhibitory synapses onto bipolar and ganglion cells, as well as other amacrine cells, with multiple microcircuit *motifs* of feedforward, feedback, lateral, and reciprocal inhibition (reviewed in (2)). Of the ~60 different types of amacrine cells that have been identified in mammalian retinas (3, 4), AII amacrine cells are the most numerous (5) and arguably also the most extensively studied (reviewed in (2)). The AII contributes to both scotopic and photopic vision by providing a crucial feedforward pathway between rod bipolar cells and ON- and OFF-cone bipolar cells and by mediating a cross-over inhibition between the ON and OFF pathways (reviewed in (6)). A recent study suggested that the AII amacrine has the most complex “interaction repertoire” of any neuron in the central nervous system (CNS), with connections to at least 28 different cell types (7).

To understand synaptic integration in AII amacrines, we need to determine the location and identity of chemical (excitatory and inhibitory) and electrical synaptic connections. Excitatory, glutamatergic input is provided by rod bipolar cells at AII arboreal dendrites (8–11) and by OFF-cone bipolar cells at AII lobular dendrites (10, 12–14). For both locations, there is evidence that the input is mediated by AMPA-type non-NMDA receptors with relatively high Ca^2+^ permeability (15–18). AII amacrines also express NMDA receptors (19–22), evidently with an exclusive extrasynaptic location (23).

In contrast to the excitatory input, much less is known about the inhibitory inputs to AII amacrines. Ultrastructural studies have provided evidence for input to AIIs from other types of amacrine cells, presumably inhibitory, at multiple locations. These include the transition between the soma and apical dendrite, the apical dendrite itself, the lobular dendrites and appendages, and the arboreal dendrites (7, 10, 11). Although little is known about the cellular identity and functional properties of these inputs, it is likely that they represent both glycinergic input from narrow-field amacrines and GABAergic input from wide-field amacrines. Whole-cell recording has provided evidence for glycinergic synaptic input to AII amacrines (24, 25), but the cellular identity of these presynaptic glycinergic neurons is as yet unknown.

Concerning potential GABAergic input to AII amacrines, there is electrophysiological evidence that GABA evokes a response with pharmacology characteristic of GABA_A_ receptors (20, 22, 24, 26). A series of studies have provided evidence for the cellular identity of putative GABAergic inputs to AII amacrines. First, light- and electron microscopy revealed synaptic specializations between processes of dopaminergic amacrine cells and the transition between the soma and apical dendrite of AIIs (27). These contacts were originally interpreted as dopaminergic synapses (27–30), but subsequent work revealed that dopaminergic amacrines may release GABA in addition to dopamine (31–33), with immunolabeling for GABA_A_ receptors at synapses between dopaminergic and AII amacrine cells (34), suggesting that the connection is GABAergic. In addition, a recent study of mouse retina with serial-section electron microscopy found extensive input from the presumably GABAergic NOS-1 amacrine cell (35) to AII arboreal dendrites (36). Consistent with this, optogenetic depolarization of NOS-1 cells evoked a GABA_A_ receptor-mediated response in AIIs (36). Despite the extensive evidence for GABAergic input to AII amacrines, little is known about the physiological and pharmacological properties and the molecular identity of the GABA_A_ receptors expressed by these cells.

In this study, we used electrophysiological recording to investigate the functional properties of AII GABA_A_ receptors. Surprisingly, but consistent with an earlier study from our laboratory (24), we did not observe spontaneous or evoked postsynaptic currents mediated by GABA_A_ receptors in AIIs. However, analysis of GABA-evoked responses in AII nucleated patches suggested the presence of GABA_A_ receptors with very slow decay kinetics and apparent single-channel conductance of ~23 pS. Together with the pharmacological properties, this suggested the presence of GABA_A_ receptors with α2 and/or α3, as well as γ2 subunits.

## Materials and methods

### Retinal slice preparation and visual targeting of neurons

General aspects of the methods have previously been described in detail (17, 24). The use of animals in this study was carried out under the approval of and in accordance with the Animal Laboratory Facility at the Faculty of Medicine at the University of Bergen (accredited by AAALAC International). Albino rats (Wistar HanTac; 4-7 weeks postnatal, male and female) were deeply anaesthetized with isoflurane in oxygen and killed by cervical dislocation. Vertical retinal slices were cut by hand and visualized using an Axioskop 2 FS (Zeiss) with a ×40 (0.75 NA) water immersion objective or a BX51WI (Olympus) with a ×40 (0.8 NA) water immersion objective, both equipped for infrared differential interference contrast (IR-DIC) videomicroscopy.

### Solutions and drug application

The standard extracellular perfusing solution was continuously bubbled with 95% O_2_ - 5% CO_2_ and had the following composition (in mM): 125 NaCl, 25 NaHCO_3_, 2.5 KCl, 2.5 CaCl_2_, 1 MgCl_2_, 10 glucose (pH 7.4). For whole-cell and nucleated-patch recordings, pipettes were filled with a solution that had the following composition (in mM): 130 KCl, 8 NaCl, 10 HEPES, 1 CaCl_2_, 5 EGTA, 4 MgATP, 2 QX-314 (pH adjusted to 7.3 with KOH). In some nucleated patch experiments, QX-314 was omitted from the pipette solution. Alexa Fluor 594 (40 or 50 µM; Invitrogen/Thermo Fisher Scientific) was included in all pipette solutions and permitted visualization of the complete cellular morphology with wide-field fluorescence microscopy after whole-cell and patch recordings.

For recordings of spontaneous inhibitory postsynaptic currents (spIPSCs), neurotransmitter receptor antagonists were added directly to the extracellular bath solution at the following concentrations (µM): 0.3 strychnine (Research Biochemicals International) to block glycine receptors; 10 6-cyano-7-nitroquinoxaline-2,3-dione (CNQX; Hello Bio) to block non-NMDA receptors; 20 (RS)-3-(2-carboxypiperazin-4-yl)-propyl-1-phosphonic acid (CPP; Hello Bio) to block NMDA receptors.

In some experiments, we attempted to evoke synaptic release of neurotransmitter by local application of extracellular solution with increased concentration of K^+^ (to depolarize putative presynaptic sources and thereby evoke neurotransmitter release). The high-K^+^ solution (in mM: 125 NaCl, 22.5 KCl, 2.5 CaCl_2_, 1 MgCl_2_, 5 HEPES, 10 glucose; pH adjusted to 7.4 with HCl) was applied by pressure (0.2 - 0.3 bar; 5 - 10 s) from a patch pipette connected to a pneumatic drug ejection system (PDES-01DDM; npi electronic). The same system was also used for pressure application (0.1 - 0.2 bar; 15 - 200 ms) of GABA (1 mM) from a patch pipette to AII amacrine cells. GABA was dissolved in a HEPES-buffered solution of the following composition (in mM): 145 NaCl, 2.5 KCl, 2.5 CaCl_2_, 1 MgCl_2_, 5 hemiNa-Hepes, and 10 glucose (pH adjusted to 7.4 with HCl).

### Fast drug application

Fast application was performed with a theta-tube pipette (septum thickness ~117 µm, final tip diameter ~300 µm; Hilgenberg) according to the description of Jonas (37), for additional details, see (17, 18). The patch was positioned near the interface between the control solution and the agonist-containing solution continuously flowing out of each barrel, about 100 µm away from the tip of the theta tube. Concentration jumps of agonist were performed by rapidly moving the application pipette and thus the interface between the two solutions. One barrel of the theta tube contained 3 mM GABA, dissolved in a HEPES-buffered solution of the same composition as used for pressure application of GABA (see above). The other barrel contained either the HEPES-buffered solution without GABA, or, alternatively, the HEPES-buffered solution with either SR95531 (3 µM; Tocris Bioscience; to block GABA_A_ receptors), ZnCl_2_ (10 or 100 µM), zolpidem (100 nM or 1 µM; Synthélabo Recherche), or 4,5,6,7-tetrahydroisoxazolo[5,4-*c*]pyridin-3-ol hydrochloride (THIP; 1 or 10 µM; Tocris Bioscience). Agonist pulses were applied at 30 or 40 s intervals. The solution exchange time was measured as the open-tip response when switching between the HEPES-buffered solution and the same solution diluted to 10% (cf. (17)). Under optimal conditions, the 20-80% rise time of the open-tip response ranged from 150 to 400 µs (10-90% rise time of 200 - 600 µs). For nucleated patches, this does not represent the true exchange time, which is expected to be slower because of the size of the patch. In experiments where we needed to switch between different solutions for a given barrel, complete exchange took 85 - 100 s. Solutions were either made up freshly for each experiment or were prepared from aliquots stored at -20°C and diluted to the final concentration on the day of the experiment.

### Electrophysiological recording and data acquisition

Patch pipettes were pulled from thick-walled borosilicate glass (outer diameter, 1.5 mm; inner diameter, 0.86 mm; Sutter Instrument). The open-tip resistance with the pipette in the bath ranged from 5 to 7 MΩ when filled with intracellular solution. Electrophysiological recordings were performed with an EPC9 dual or an EPC10 quadro amplifier controlled by Patchmaster software (HEKA Elektronik). For details of electrophysiological recording, see (38).

Nucleated-patch recordings were obtained after establishing the whole-cell configuration, by slowly withdrawing the pipette and applying continuous light suction (~50 mbar). When a nucleated patch was successfully isolated, the reduced membrane capacitance resulted in current transients that were canceled by re-adjustment of the amplifier *C*_slow_ neutralization circuitry. For nucleated patch recordings, signals were low-pass filtered (analog 3- and 4-pole Bessel filters in series) with a corner frequency (-3 dB) at 1/5 of the inverse of the sampling interval (typically 50 µs). For whole-cell recordings, signals were low-pass filtered at 2 kHz and the sampling interval was 100 µs. All recordings were carried out at room temperature (22 - 25°C). The data acquisition software corrected all holding potentials for liquid junction potentials on-line. Theoretical liquid junction potentials were calculated with JPCalcW (Axon Instruments/Molecular Devices).

### Electrophysiological data analysis

Data were analyzed with Fitmaster (HEKA Elektronik), IGOR Pro (WaveMetrics), and Excel (Microsoft). Before averaging of nucleated-patch responses, individual response waveforms were aligned at the point of steepest rise. The peak amplitude of GABA-evoked currents in patches was measured after averaging (typically 5 - 40 repetitions) and baseline subtraction. The decay time-course of averaged GABA responses was estimated by curve fitting with exponential functions. For single-exponential functions, we used the function:


(1) $$I\left(t\right)=A\times e^{\left(-t/\tau \right)}+I_{ss}$$

where *I*(t) is the current as a function of time, *A* is the amplitude at time 0, *τ* is the time constant, and *I_ss_* is the steady-state amplitude. For double-exponential functions, we used the function:


(2) $$I\left(t\right)=A_{1}\times e^{\left(-t/\tau_{1}\right)}+A_{2}\times e^{\left(-t/\tau_{2}\right)}+I_{ss}$$

where *I*(t) is the current as a function of time, *A*_1_ and *A*_2_ are the amplitudes at time 0 of the first and second exponential components, τ_1_ and τ_2_ are the time constants of the first and second exponential components, and *I*_ss_ is the steady-state amplitude. For triple-exponential functions, a third component $A_{3}\times e^{\left(-t/\tau_{3}\right)}$ was added to eqn (2) above. Fitting was generally started 200 - 600 µs after the peak amplitude. For double- and triple-exponential functions, the amplitude contribution of a given component *A*_x_ (*A*_1_, *A*_2_ or *A*_3_) was calculated as 100% × (*A*_x_/(*A*_1_+*A*_2_)) or 100% × (*A*_x_/(*A*_1_+*A*_2_+*A*_3_)), respectively. As the relative amplitude of the exponential components depends on the location of time 0, we defined the start of the response as the point in time at which the current rose from the baseline noise (determined by eye). For double- and triple-exponential fits, weighted time constants were calculated as the sum of the individual time constants multiplied by the relative amplitude contribution of the corresponding component.

For illustration purposes, most raw data records were low-pass filtered (-3 dB; digital non-lagging Gaussian filter at 0.5 - 2 kHz). Unless otherwise noted, the current traces shown in the figures represent individual traces.

### Non-stationary noise analysis

We applied non-stationary noise analysis to patch responses evoked by brief pulses (2 ms for six patches; 5 ms for one patch) of GABA (3 mM) to estimate apparent single-channel conductance and open probability of the receptor channels, for details, see (39). The ensemble mean response was binned and variance versus mean curves were plotted for the decay phase (i.e., the interval between the peak response and the end of the decay phase) and fitted with the parabolic function:


(3) $$\sigma^{2}(I)=iI-I^{2}/N+\sigma_{b}^{2}$$

where *σ^2^* (I) is the variance as a function of mean current, *i* is the apparent single-channel current, *N* corresponds to the number of available channels in the patch and $\sigma_{b}^{2}$ is the variance of the background noise. The apparent single-channel chord conductance (γ) was calculated as:


(4) $$\gamma =i/(V_{m}-E_{rev})$$

with a holding potential (*V*_m_) of -60 mV and the reversal potential (*E*_rev_) set to 0 mV (for a recording condition with symmetrical Cl^-^ concentration). The open probability (*P*_open_) was calculated using the equation:


(5) $$P_{open}=I/iN$$

where *i* is the apparent single-channel current, *I* is the mean current, and *N* is the number of available channels in the patch.

### Wide-field fluorescence microscopy

All cells (both whole-cell and patch recordings) were imaged with wide-field fluorescence microscopy after recording to confirm the identity of the cell. For some cells we acquired a series of fluorescence images at closely spaced focal planes using a digital CCD camera, either a CoolSnap ES (Photometrics/Roper Scientific) controlled by µManager software (www.micro-manager.org) or an Imago QE controlled by TILLvisION software (TILL Photonics), for details see (40). Subsequently, images were processed with Huygens (64 bit, Windows; Scientific Volume Imaging) to remove noise and reassign out-of-focus light by deconvolution with a theoretical point-spread function (CMLE method). Huygens was also used to generate maximum intensity projections and to adjust contrast, brightness, and gamma (identical settings for the entire image).

### Statistical analysis

Data are presented as mean **±** SD (*n* = number of cells, patches or responses; as indicated) with ranges either displayed by individual data points in bar graphs or stated explicitly. Percentages are calculated as percentage of control. Statistical analyses with comparisons between or within groups were performed using Student’s two-tailed *t* test (unpaired, paired or ratio paired; as indicated) with Prism (GraphPad Software). Differences were considered statistically significant at the *P* ≤ 0.05 level.

## Results

### Targeting and identification of AII amacrine cells in rat retinal slices

**Figure 1:**
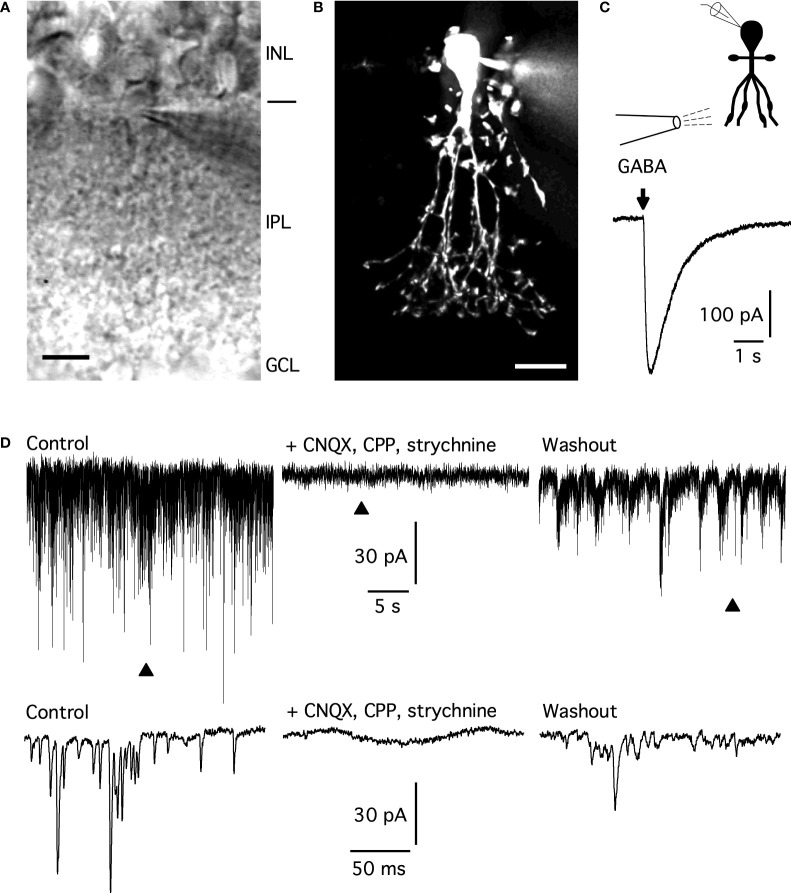
Identification of AII amacrines and lack of spontaneous GABA receptor-mediated synaptic currents. **(A)** Infrared differential interference contrast videomicrograph (IR-DIC) of a retinal slice with visible cell body and apical dendrite of an AII amacrine cell during whole-cell recording (note visible tip of patch pipette). Scale bar, 10 µm. Retinal layers indicated by abbreviations (INL, inner nuclear layer; IPL, inner plexiform layer; GCL, ganglion cell layer). **(B)** Maximum intensity projection of image stack of wide-field fluorescence image (after deconvolution) of AII amacrine in **(A)** filled with Alexa 594 *via* patch pipette. Scale bar, 10 µm. **(C)** Current evoked in an AII amacrine (*V*_hold_ = -60 mV) by application of GABA (1 mM, 100 ms) from a puffer pipette during whole-cell, voltage-clamp recording (the recording configuration is indicated by the schematic at top, with the tip of the puffer pipette located approximately over stratum 5 of the inner plexiform layer). The arrow indicates the onset of GABA application. The bath and puffer pipette solutions contained CNQX (10 µM) and strychnine (300 nM). Voltage-gated Na^+^ channels were blocked by QX-314 in the intracellular solution. **(D)** Whole-cell, voltage-clamp recording from an AII amacrine (*V*_hold_ = -60 mV) in three different conditions (the intracellular solution contained QX-314). Left: control (no pharmacological blockers of synaptic receptors) with relatively high frequency of (inward) spontaneous postsynaptic currents (spPSCs). Middle: with CNQX (10 µM), CPP (20 µM), and strychnine (300 nM) in the bath solution, no spPSCs were observed. Right: after CNQX, CPP, and strychnine were washed out, the amplitude and frequency of spPSCs partially recovered. For each condition, current is displayed at both a slow (top) and a fast (bottom) time scale, triangles mark approximate location of epochs expanded at bottom.

AII amacrine cells in retinal slices were targeted for recording based on the following criteria: a cell body located at the border of the inner nuclear layer and the inner plexiform layer, a thick apical dendrite descending into the inner plexiform layer (**Figure 1A**), and a characteristic pattern of inward action currents following 5-mV depolarizing voltage pulses (5-ms duration) from *V*_hold_ = -60 mV (cf. (15)). After the AII was filled with dye, fluorescence microscopy revealed the distinct morphology with large lobular appendages in the distal region of the inner plexiform layer and thin arboreal dendrites ramifying in the proximal region of the inner plexiform layer (**Figure 1B**).

### AII amacrine cells respond to GABA

To verify that AII amacrine cells respond to GABA during our recording conditions, we performed whole-cell, voltage-clamp recordings in retinal slices and applied GABA (1 mM) by pressure from a puffer pipette positioned close to the surface of the slice. The tip of the pipette was located in the proximal part of the inner plexiform layer, directed towards the region with the distal arboreal dendrites of the AII (**Figure 1C**). With the intracellular and bath solutions used, *E*_Cl_ was close to 0 mV and the cells were voltage-clamped at *V*_hold_ = -60 mV. Both the bath solution and the solution in the puffer pipette contained CNQX (10 µM) to block ionotropic non-NMDA receptors and strychnine (300 nM) to block glycine receptors. For the cell illustrated in **Figure 1C**, a brief (100 ms) puff of GABA evoked a large, transient inward current (peak amplitude ~325 pA). Robust responses were obtained for multiple locations of the pipette tip across the inner plexiform layer. Similar results were obtained for a total of five AII amacrines and confirm earlier observations reported for AII cells in rat and rabbit retina (20, 24, 26), suggesting that AIIs express ionotropic GABA receptors.

### No GABAergic spontaneous postsynaptic currents in AII amacrine cells

To investigate potential GABAergic synaptic inputs to AII amacrine cells, we performed whole-cell, voltage-clamp recordings in retinal slices (**Figure 1D**; *V*_hold_ = -60 mV). When the bath solution contained no blockers of neurotransmitter receptors, we observed a high frequency of spontaneous inward currents with fast kinetics (*E*_Cl_ ~ 0 mV). In this condition, both excitatory currents (mediated by non-selective cation channels) and inhibitory currents (mediated by chloride channels) will appear as inward currents. From earlier investigations, there is strong evidence for both glutamatergic and glycinergic spontaneous postsynaptic currents (spPSCs) in AII amacrine cells (16, 17, 24, 25). After recording for 2 - 3 min in this control condition, we changed to a bath solution containing CNQX (10 µM), CPP (20 µM; to block NMDA receptors), and strychnine (300 nM). In this condition, spontaneous chemical synaptic activity in AII amacrines was completely blocked (**Figure 1D**; *n* = 5 cells), suggesting that there were no GABAergic spIPSCs. The recording period in pharmacological blockers was 4 - 8 min to ensure that even low-frequency events could be detected. When the bath solution was changed back to control without neurotransmitter receptor antagonists, the spontaneous synaptic activity partially recovered (**Figure 1D**).

### High-K^+^ stimulation fails to evoke GABAergic postsynaptic currents in AII amacrine cells

Given the morphological evidence for putative GABAergic synaptic input to AIIs from dopaminergic amacrine cells (34) and NOS-1 amacrine cells (36), the lack of GABAergic spIPSCs is surprising, but consistent with earlier observations from our laboratory (24). However, if we assume that the processes of dopaminergic and NOS-1 amacrine cells presynaptic to AIIs contain synaptic vesicles with GABA, it might be possible to evoke synaptic release without direct manipulation of the presynaptic neurons.

**Figure 2:**
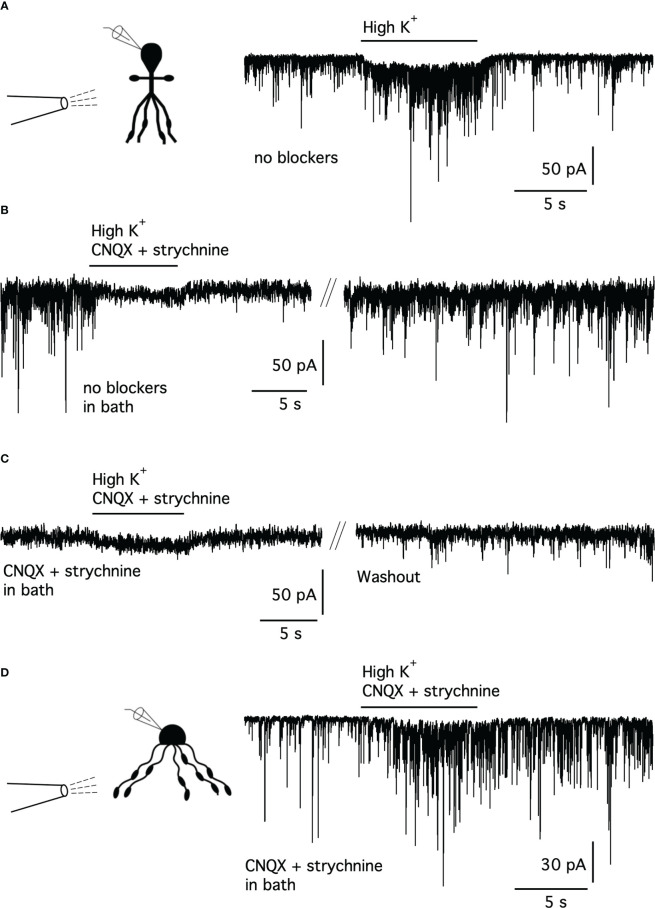
High-K^+^ stimulation of synaptic release from presynaptic terminals does not evoke synaptic currents mediated by GABA receptors in AII amacrine cells. **(A)** Puffer pipette application of high-K^+^ solution (HEPES-buffered extracellular solution with [K^+^] increased from 2.5 to 22.5 mM) onto an AII amacrine (*V*_hold_ = -60 mV, *E*_Cl_ ~ 0 mV) evoked a marked increase in postsynaptic currents (PSCs). Here and in **(D)**, experimental configuration and identity of cell from which the recording was made indicated by the schematic (inset, left). No blockers present in puffer pipette solution or in bath. Here and later, duration of application indicated by horizontal bar above current trace. **(B)** When high-K^+^ puffer pipette solution with CNQX (10 µM) and strychnine (300 nM) was applied to an AII amacrine cell (different cell than in **(A)**; no blockers in bath), all PSCs were blocked (left). After a short period of washout (~20 s; marked by parallel lines), the PSCs fully recovered (right). **(C)** When application of high-K^+^ solution with CNQX and strychnine to the AII amacrine cell (same as in **(B)**) was repeated after adding CNQX (10 µM) and strychnine (300 nM) to the bath solution, PSCs were blocked both before and during application. In this condition, application of high-K^+^ solution evoked a small inward current, probably corresponding to the local depolarizing shift of *E*_K_. After a period of washout (~10 min; marked by parallel lines), the PSCs partially recovered (right). **(D)** Recording from an A17 amacrine cell (*V*_hold_ = -70 mV, *E*_Cl_ ~ 0 mV). High-K^+^ solution (with 10 µM CNQX and 300 nM strychnine in both puffer pipette and bath) evoked an increase of PSCs. Note that the increased frequency of PSCs outlasted the period of application of high-K^+^ solution.

To evoke release of synaptic vesicles, we applied an extracellular solution with high K^+^ concentration from a puffer pipette placed at the inner plexiform layer, designed to evoke depolarization and trigger activation of voltage-gated Ca^2+^ channels. Relative to the control solution, the high-K^+^ solution increased [K^+^] from 2.5 to 22.5 mM, thereby changing *E*_K_ from -102 to -45 mV (for a temperature of 25˚C). The high-K^+^ solution was applied approximately once every minute. As a first step, we applied the high-K^+^ solution to AII amacrine cells in the absence of any pharmacological blockers in the bath or puffer solutions. As illustrated in **Figure 2A**, application of high-K^+^ solution (8-s duration) increased the frequency of PSCs. This effect was robust and with repeated application could be observed for >20 min.

Next, to pharmacologically isolate potential GABA-mediated currents, the recordings were performed in the presence of CNQX and strychnine. The AII amacrine cell illustrated in **Figures 2B, C** displayed robust activity with spPSCs before application of the puffer pipette solution (**Figure 2B**, left trace). When we puffed high-K^+^ with CNQX and strychnine in the pipette solution, but without the same blockers in the bath, all PSCs were completely blocked (**Figure 2B**, left trace). After washout of the puffer pipette solution, the activity of spPSCs recovered quickly (**Figure 2B**, right trace). This suggested that the high-K^+^ solution was unable to evoke any GABAergic PSCs. The same conclusion was reached when we repeated the application of high K^+^ with CNQX and strychnine in both the pipette and bath solution (**Figure 2C**, left trace). The spPSCs recovered slowly (and incompletely) when the bath solution was changed back to control without neurotransmitter receptor antagonists (**Figure 2C**, right trace). With all spPSCs blocked by CNQX and strychnine in the bath, the effect of puffing high-K^+^ solution was observed as a small, inward current during application (**Figure 2C**, left trace), likely to reflect a more depolarized *E*_K_ and the accompanying depolarization of other cells electrically coupled to the recorded cell (41, 42). The failure to evoke PSCs was observed both when the puffer pipette tip was placed in stratum 5 (S5; to activate putative inputs from the NOS-1 amacrine cells) (36) or in S1-S3 (to activate putative inputs from dopaminergic amacrine cells) (34). Similar results, with no evidence for GABAergic IPSCs, were observed for a total of seven AII amacrine cells.

As a control, when high-K^+^ solution was applied to A17 amacrine cells under the same recording conditions (with CNQX and strychnine in both the puffer pipette and bath), we consistently observed a marked increase in the frequency of PSCs (**Figure 2D**; *n* = 5 cells). Both the spontaneous PSCs (observed before puffing high-K^+^) and the evoked PSCs (observed during puffing high-K^+^) are likely to be GABAergic (cf. (38)).

### GABA-evoked responses of AII nucleated patches are mediated by GABA_A_ receptors

**Figure 3:**
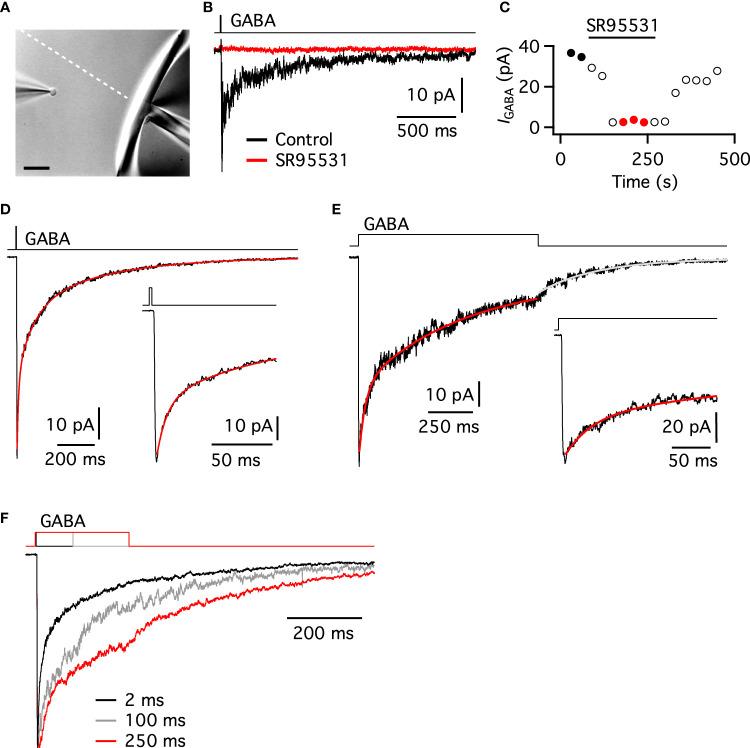
Deactivation and desensitization kinetics of GABA_A_ receptors in nucleated patches of AII amacrine cells. **(A)** IR-DIC image of AII nucleated patch positioned close to the liquid filament interface (indicated by the dashed white line) formed by the two solutions flowing out of a theta-tube application pipette. Scale bar, 30 µm. **(B)** Current responses of a nucleated patch (*V*_hold_ = -60 mV, *E*_Cl_ ~ 0 mV) from an AII amacrine evoked by a brief (5 ms) pulse of GABA (3 mM) from a theta-tube pipette (lower black trace, average of two trials). The response was blocked after exposing the patch to the selective GABA_A_ receptor antagonist SR95531 (3 µM; red trace, average of three trials). Here and later, the black line above the current responses corresponds to the square-wave voltage pulse (from the digital-to-analog output of the interface of the patch-clamp amplifier) used to drive the piezo actuator with the theta-tube pipette. Note that this waveform precedes the patch response by a few ms. **(C)** Peak amplitude of GABA-evoked (5 ms, 3 mM) currents obtained at 30 s intervals (same nucleated patch as in **(B)**). Note the reversible block of GABA-evoked responses during exposure to SR95531 (3 µM). The data points marked by filled black circles and red circles correspond to the responses used to calculate average waveforms in **(B)**. **(D)** Current response (lower black trace; average of 36 trials) of an AII nucleated patch to a brief (~2 ms) pulse of GABA (3 mM), overlaid with triple-exponential fit to decay phase corresponding to deactivation (red). Inset shows the early phase of the response at an expanded time scale. **(E)** Current response (lower black trace; average of nine trials) of an AII nucleated patch to a long (1 s) pulse of GABA (3 mM), overlaid with double-exponential fit to desensitization decay phase (red) and single-exponential fit to deactivation decay phase following end of GABA pulse (gray). Inset shows the early phase of the response at an expanded time scale. **(F)** Normalized current responses of AII nucleated patch to a series of applications of GABA (3 mM) of variable duration (~2 ms, black trace, average of 30 trials; 100 ms, gray trace, average of seven trials; 250 ms, red trace, average of eight trials). Note the difference between the slower desensitization and the faster deactivation kinetics, including the transition from desensitization to deactivation for the two longer pulse durations.

For investigating the kinetic properties of the GABA receptors, ultrafast drug application to outside-out patches is the preferred method, as it can mimic the conditions inside a synaptic cleft (37). In contrast, puffing GABA onto a cell in the whole-cell configuration is unable to achieve the required speed of application. Accordingly, we first attempted to obtain responses using conventional outside-out patches and ultrafast application of GABA. However, the number of patches with responses that were sufficiently large for analysis was very low, with most patches displaying no or only very small responses. This suggested that the distribution of GABA_A_ receptors at the cell body of an AII is markedly heterogenous, potentially with small and infrequent areas of clustered receptors and larger areas with no or very few receptors. This is very different from the results for AMPA-type glutamate and glycine receptors, where we obtained a considerably higher success rate, often with relatively large responses, for conventional outside-out patches isolated from the cell body (17, 18, 24). Because of the very low success rate of obtaining adequate GABA responses with conventional outside-out patches, we instead used nucleated patches that consistently displayed robust responses (**Figure 3A**).

An example of GABA-evoked responses obtained with a nucleated patch is illustrated in **Figure 3B**. When this nucleated patch was exposed to a brief (~5 ms) pulse of GABA (3 mM), it displayed a transient inward current with fast rise time and slower decay (*V*_hold_ = -60 mV, *E*_Cl_ ~ 0 mV). With repeated application of GABA (30 s intervals), we observed response rundown (**Figure 3C**). When the specific GABA_A_ receptor antagonist SR95531 (3 µM) was added to the solution in the control barrel of the theta tube, the GABA-evoked response was completely blocked (**Figures 3B, C**). After washing out SR95531, the GABA-evoked response recovered quickly (**Figure 3C**). Similar results, with complete block of GABA-evoked responses by SR95531, were observed for a total of three nucleated patches. This strongly suggested that the responses were mediated by GABA_A_ receptors.

### Kinetics of GABA_A_ receptors in AII nucleated patches

Because it is not possible to obtain ultrafast application and near-synchronous activation of all receptors using nucleated patches with a theta-tube application system, the activation and deactivation kinetics may appear somewhat slower for nucleated than for conventional outside-out patches. For the nucleated patch illustrated in **Figure 3D**, a brief (~2 ms) pulse of GABA (3 mM) evoked a response that rose rapidly to a peak, with 20-80% rise time of 809 µs (10-90% rise time 1242 µs) and peak amplitude of 63 pA (average of 36 trials). For a total of 11 nucleated patches tested in this way, the average peak amplitude was 47.3 ± 19.7 pA (range 19.9 - 81.1 pA). The average 20-80% rise time was 913 ± 212 μs (range 635 - 1297 μs; average 10-90% rise time 1465 ± 377 µs, range 975 - 1962 µs). At the end of the 2 ms pulse, the response decayed with a very slow time course (**Figure 3D**). This decay corresponds to deactivation, i.e., the closing of channels after removal of agonist and provides information about the gating properties of the receptors. Adequately fitting the decay time course required a triple-exponential function, with τ_1_ = 8.4 ms, τ_2_ = 84.6 ms, and τ_3_ = 418 ms (with amplitude contributions of 45, 30 and 25%, respectively). The weighted deactivation time constant (τ_w_) was 133 ms. For the 11 nucleated patches tested with brief pulses of GABA, the average τ_1_ was 9.8 ± 4.0 ms (range 4.3 - 17.5 ms; 41 ± 13% amplitude contribution), the average τ_2_ was 96.1 ± 38.5 ms (range 63.8 - 173.8 ms; 35 ± 10% amplitude contribution), and the average τ_3_ was 523 ± 116 ms (range 378 - 779 ms; 25 ± 9% amplitude contribution). The average τ_w_ of deactivation was 163 ± 39 (range 103 - 229 ms). The most important experimental observables and response parameters have been summarized in **Table 1**.

**Table 1:** Experimental measurements for GABA_A_ receptors of AII amacrine cells in rat retina.

Observable	Mean ± SD (range) *n* = number of patches
Peak amplitude (pA), 2 ms pulse	47.3 + 19.7 pA (19.9 - 81.1) *n* = 11
20-80% rise time (µs), 2 ms pulse	913 ± 212 (635 - 1297) *n* = 11
10-90% rise time (µs), 2 ms pulse	1465 ± 377 (975 - 1962) *n* = 11
Deactivation τ_1_ (ms), 2 ms pulse rel. contribution (%)	9.8 ± 4.0 (4.3 - 17.5) 41 ± 13% *n* = 11
Deactivation τ_2_ (ms), 2 ms pulse rel. contribution (%)	96.1 ± 38.5 (63.8 - 173.8) 35 ± 10% *n* = 11
Deactivation τ_3_ (ms), 2 ms pulse rel. contribution (%)	523 ± 116 (378 - 779) 25 ± 9% *n* = 11
Deactivation τ_w_ (ms), 2 ms pulse	163 ± 39 (103- 229) *n* = 11
Desensitization τ_fast_ (ms), 1 s pulse rel. contribution (%)	52.5 ± 16.6 (37.1 - 83.6) 45 ± 14% *n* = 7
Desensitization τ_slow_ (ms), 1 s pulse rel. contribution (%)	557 ± 157 (306 - 799) 55 ± 14% *n* = 7
Desensitization τ_w_ (ms), 1 s pulse	(208 - 536) *n* = 7
Deactivation τ (ms), following desensitization after 1 s pulse	537 ± 159 (329 - 798) *n* = 7
*P*_o, max_ from non-stationary noise analysis	0.56 ± 0.06 (0.47 - 0.63) *n* = 7
Single-channel conductance (γ) from non-stationary noise analysis (pS)	23.2 ± 2.8 (20.4 - 27.1) *n* = 7
Mean number of available channels from non-stationary noise analysis	68.2 ± 29 (26.5 - 109.9) *n* = 7

Electrophysiological data were obtained with voltage-clamp recordings from nucleated patches from AII amacrine cells in retinal slices. GABA (3 mM) was applied with a fast perfusion system (See Materials and methods).

To study the time course of desensitization, i.e., the closure of channels in the maintained presence of agonist, we applied longer (1 s) pulses of GABA (3 mM). For the nucleated patch illustrated in **Figure 3E**, the GABA-evoked response (average of nine trials) displayed a peak amplitude of 83 pA and a 20-80% rise time of 976 µs (10-90% rise time of 1721 µs). During the prolonged exposure to GABA, there was pronounced desensitization of the response (**Figure 3E**). The time course of desensitization could be well fitted by a double-exponential function, with τ_fast_ = 37.2 ms and τ_slow_ = 578 ms (with amplitude contributions of 40 and 60%, respectively). The τ_w_ of desensitization was 361 ms. For a total of seven patches tested in this way, the average τ_fast_ was 52.5 ± 16.6 ms (range 37.1 - 83.6 ms; 45 ± 14% amplitude contribution) and the average τ_slow_ was 557 ± 157 ms (range 306 - 799 ms; 55 ± 14% amplitude contribution). The average τ_w_ of desensitization was 333 ± 120 ms (range 208 - 536 ms).

For GABA_A_ receptors, there is evidence that desensitization can slow the subsequent time course of deactivation (43, 44) and shift receptors into a high-affinity state (45). Following removal of GABA at the end of a longer pulse of GABA, we could directly observe the change from desensitization to deactivation. For the nucleated patch illustrated in **Figure 3E**, the time course of deactivation (after removal of GABA) could be well fitted with a single exponential function with a time constant of 359 ms. For a total of seven patches tested with 1-s pulses of GABA (3 mM), the time course of deactivation following desensitization was well fitted with a single exponential function, with an average time constant of 537 ± 159 ms (range 329 - 798 ms). This post-desensitization deactivation was much slower than the brief-pulse (~2 ms) deactivation (with τ_w_ = 163 ± 39 ms; *P*< 0.0001, unpaired *t* test). In contrast, there was no statistically significant difference between the post-desensitization deactivation and the τ_w_ of desensitization (for a 1-s pulse of GABA) for these patches (333 ± 120 ms; *P* = 0.2106, paired *t* test).

For two patches we obtained GABA-evoked responses to multiple pulse durations, allowing us to directly compare the difference between deactivation and desensitization. For the nucleated patch illustrated in **Figure 3F**, the responses evoked by 2-, 100-, and 250-ms pulses of GABA (3 mM) have been overlaid after normalization to the peak amplitudes. At the end of the 100- and 250-ms pulses, the difference in decay time course can be readily observed during the change from relatively slow desensitization to faster deactivation. For this nucleated patch, the τ_w_ of deactivation following a brief (~2 ms) pulse was 192 ms. After the 100 ms pulse, the time constant of deactivation was 393 ms and after the 250 ms pulse it was 469 ms (single-exponential fits). Similar results were obtained for the other nucleated patch. These results suggested that increasing desensitization of the AII GABA_A_ receptors slows the subsequent deactivation. If the properties we have observed for the receptors in patches are representative for receptors that mediate potential GABAergic synaptic input to AIIs, these results suggest that the synaptic receptors have remarkably slow kinetics.

### Non-stationary noise analysis of GABA_A_ receptor-mediated responses in nucleated patches

**Figure 4:**
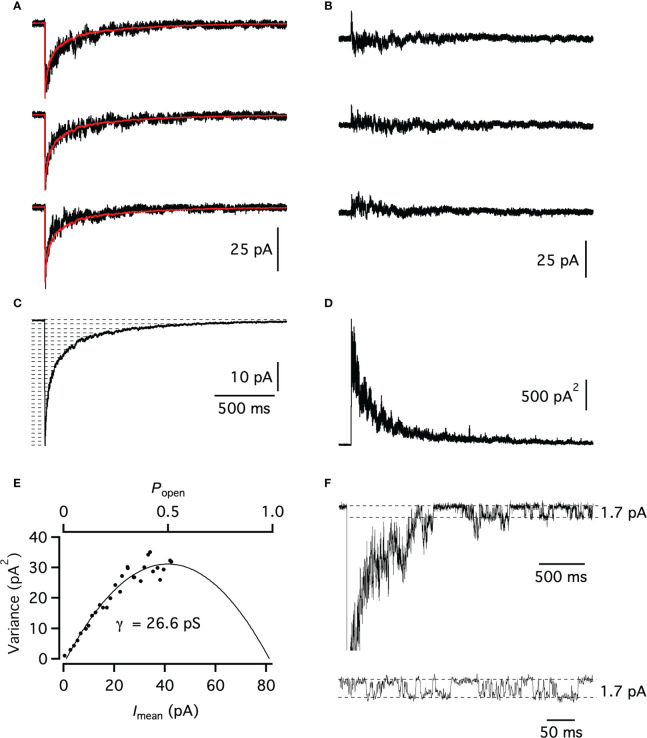
Non-stationary noise analysis of GABA-evoked responses in an AII nucleated patch. **(A)** Three individual records obtained by brief (~2 ms) pulses of GABA (3 mM) to an AII nucleated patch (*V*_hold_ = -60 mV, *E*_Cl_ ~ 0 mV) with the ensemble mean current (average of 47 trials) overlaid (red). **(B)** Associated difference currents generated by subtracting the ensemble mean current from the individual responses in **(A)**. **(C)** Mean current of all GABA-evoked responses in the ensemble. Broken horizontal lines indicate amplitude intervals used for binning mean current and variance (see Materials and methods). **(D)** Ensemble current variance (without binning) for the GABA-evoked responses, calculated from the difference currents (as in **(B)**). **(E)** Plot of the ensemble current variance **(D)** versus mean current (**(C)**; after binning). Time period used for the variance versus mean plot corresponds to data points from the peak to the end of the decay phase of the mean waveform. The data points were fitted with eqn (3). **(F)** Current response evoked by a brief (~2 ms) pulse of GABA (3 mM) to an AII nucleated patch. The peak of the inward current has been truncated for better visualization of GABA-evoked single-channel gating during the late decay phase. Inset (bottom) shows single-channel gating displayed at an expanded time scale. Broken lines indicate baseline current and inward current during channel opening (as indicated).

To estimate the single-channel conductance and the maximum *P*_open_ of the GABA_A_ receptor channels in AII nucleated patches, we applied non-stationary noise analysis. Responses were evoked by application of brief (~2 ms) pulses of GABA (3 mM). **Figure 4A** shows three individual responses evoked by GABA in the same patch, together with the superimposed ensemble mean response (*n* = 47 epochs; **Figure 4C**). The corresponding differences between each individual response and the ensemble mean response (**Figure 4B**) were used to calculate the ensemble variance (**Figure 4D**). The variance *versus* mean plot (corresponding to the decay phase after the peak response) displayed a (partial) parabolic shape (**Figure 4E**) and was fitted by the parabolic function of eqn (3). From the curve fitting, we obtained an apparent single-channel current of 1.6 pA, corresponding to an apparent single-channel chord conductance of 26.6 pS (assuming *E*_rev_ = 0). The number of available channels in the patch was estimated as 51.6, corresponding to a maximum *P*_open_ at the peak response of 0.52 (**Figure 4E**). For seven patches tested with GABA in this way, the mean single-channel chord conductance was 23.2 ± 2.8 pS (range 20.4 - 27.1 pS) and the mean number of available channels was 68.2 ± 29.0 (range 26.5 - 109.9). The average maximum *P*_open_ was 0.56 ± 0.06 (range 0.47 - 0.63). It is possible that the relatively low value for maximum *P*_open_ is caused by the slower exchange rate obtained when working with larger nucleated patches compared to smaller, conventional outside-out patches (see (15) for an analysis focused on AMPA-type receptors of AII amacrine cells). Thus, the maximum *P*_open_ for synaptic receptors of the same kind could be somewhat higher.

### Direct observations of single-channel gating in nucleated-patch responses

The estimates from non-stationary noise analysis are likely to represent weighted averages of different conductance levels of different channels or different sub-conductance states of the same types of channels. For several nucleated patches with low noise levels, discrete transitions between open and closed states could be observed during the late decay phase of individual responses (**Figure 4F**). We obtained a total of 63 measurements of single-channel openings from seven different patches (*n* = 9 measurements per patch). The current amplitudes ranged between 1.2 and 2.1 pA (corresponding to 19.3 - 35.3 pS), yielding an average single-channel chord conductance of 25.8 ± 1.9 pS, similar to the average conductance of ~23 pS obtained from non-stationary noise analysis.

### Pharmacological evidence for the presence of α2 or α3 and γ2 subunits in GABA_A_ receptors of AII nucleated patches

To investigate the subunit composition of the GABA_A_ receptors in AII nucleated patches, we made use of pharmacological compounds that have specific actions on individual GABA_A_ receptor subunits and/or specific subunit combinations. For these experiments, we applied brief pulses (5 ms) of GABA (3 mM) at 30-s intervals to nucleated patches, first in control condition and then after exposing the patch to the pharmacological compound at the desired concentration.

**Figure 5:**
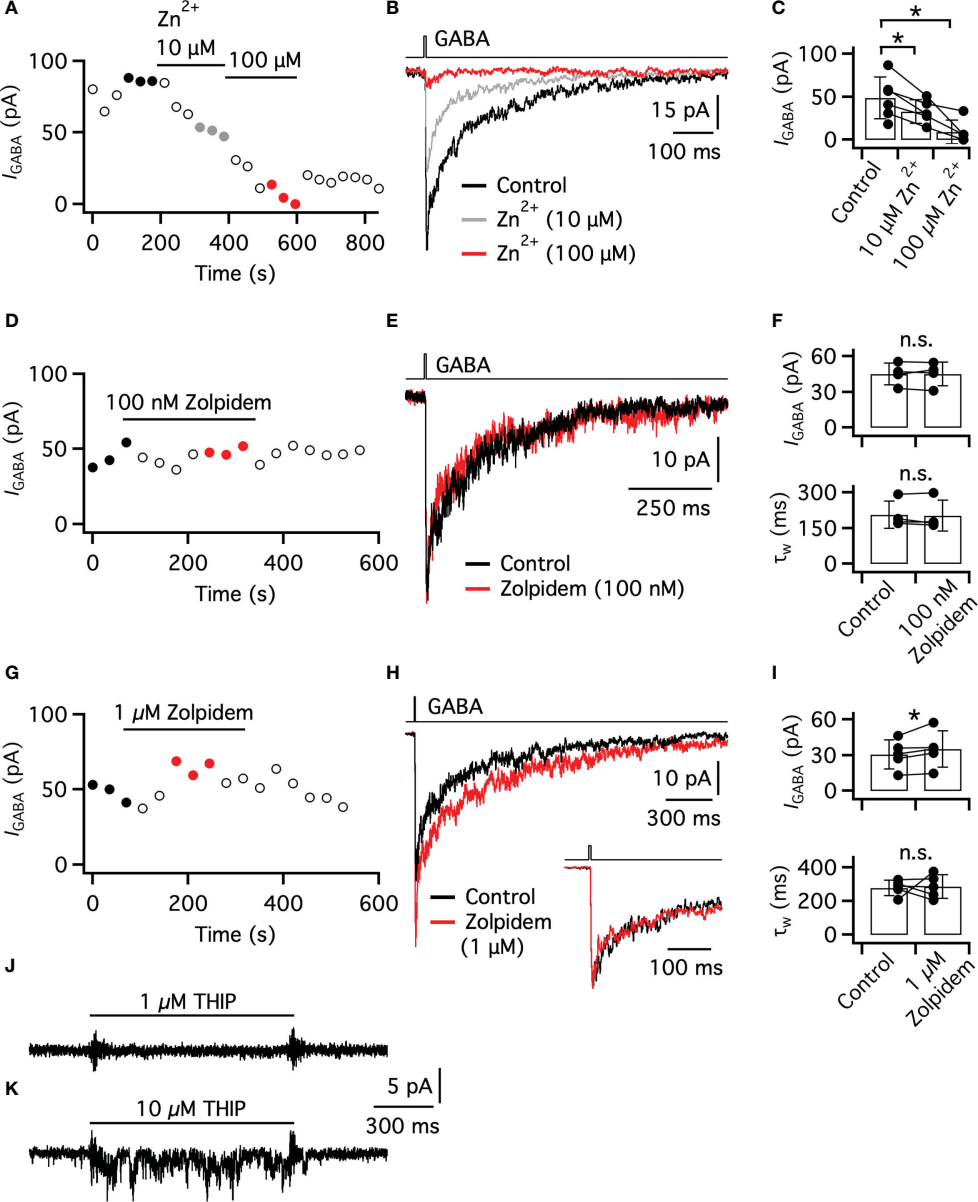
Pharmacology of GABA_A_ receptors in nucleated patches of AII amacrine cells. **(A)** Peak amplitude of GABA-evoked (5 ms, 3 mM) currents in an AII nucleated patch (30 s intervals). Note suppression of the GABA-evoked response during exposure of the patch to Zn^2+^ (10 and 100 µM). The data points marked by filled black circles, gray circles, and red circles correspond to the responses used to calculate average waveforms in **(B)**. **(B)** Responses evoked by GABA (same patch as in **(A)**) in control (black trace), during exposure to 10 µM Zn^2+^ (gray trace) and during exposure to 100 µM Zn^2+^ (red trace). Each trace is the average of three successive trials (indicated in **(A)**. **(C)** Bar graphs of peak amplitude of GABA-evoked responses (as in **(A, B)**) of AII nucleated patches (*n* = 5 patches) in control and during exposure to Zn^2+^ (10 and 100 µM). Here and later, bars represent mean ± SD and data points for the same patch are connected by lines. Statistical comparisons between averages: n.s. no significant difference (P > 0.05); * P ≤ 0.05. **(D)** Peak amplitude of GABA-evoked (5 ms, 3 mM) currents in an AII nucleated patch (30 s intervals). Note no change in peak amplitude during exposure to 100 nM zolpidem. Here and in **(G)**, the data points marked by filled black circles and red circles correspond to the responses used to calculate average waveforms in **(E, H)**, respectively. **(E)** Responses evoked by GABA (same patch as in **(D)**) in control (black trace) and during exposure to 100 nM zolpidem (red trace). Each trace is the average of three successive trials (indicated in **(D)**). **(F)** Bar graphs of peak amplitude and weighted decay time constant (τ_w_) of GABA-evoked responses (as in **(D, E)**) of AII nucleated patches (*n* = 4 patches) in control and during exposure to 100 nM zolpidem. **(G)** Peak amplitude of GABA-evoked (5 ms, 3 mM) currents in an AII nucleated patch (30 s intervals). Note moderately increased peak amplitude during exposure to 1 µM zolpidem. **(H)** Responses evoked by GABA (same patch as in **(G)**) in control (black trace) and during exposure to 1 µM zolpidem (red trace). Each trace is the average of three successive trials (indicated in **(G)**). Inset shows the early phase of the responses (normalized by peak amplitude) at an expanded time scale to facilitate comparison of the decay kinetics in the two conditions. **(I)** Bar graphs of peak amplitude and τ_w_ of GABA-evoked responses (as in **(G, H)**) of AII nucleated patches (*n* = 4 patches) in control and during exposure to 1 µM zolpidem. **(J, K)** Currents (single trials) of two different AII nucleated patches during application of THIP (1 and 10 µM, 1 s), a GABA_A_ receptor agonist with high selectivity for receptors containing the δ subunit. Note that 1 µM THIP did not evoke a response, but that 10 µM THIP evoked an increase of membrane noise and a small inward current.

Zn^2+^ acts as an antagonist at GABA_A_ receptors, with the magnitude of antagonism dependent on the specific GABA_A_ receptor subunits present (46). From studies of heterologously expressed GABA_A_ receptors, the IC_50_ for Zn^2+^ has been reported as 88 nM for αβ-containing receptors, 6 - 16 µM for αβδ-containing receptors, and 50 - 100 µM for αβγ-containing receptors. In addition, if the α subunit is of the α1 type (e.g., α1βγ), the IC_50_ for Zn^2+^ increases to ~300 µM (46, 47). For the nucleated patch illustrated in **Figures 5A, B**, GABA was applied repeatedly, first in control and then after exposing the patch to Zn^2+^ at concentrations of 10 and 100 μM. Both concentrations of Zn^2+^ evoked a clear suppression of the GABA-evoked response, as illustrated for the time series of peak amplitudes in **Figure 5A**. After washing out Zn^2+^, we observed a brief period with partial recovery. To compare the response suppression in more detail, we averaged three successive responses for each condition (control, 10 µM, and 100 µM Zn^2+^) and overlaid the waveforms (**Figure 5B**). The peak amplitude of the average response was reduced from 87 pA in control to 51 pA in 10 µM Zn^2+^ and to 6 pA in 100 µM Zn^2+^. For a total of five patches, 10 µM Zn^2+^ reduced the GABA-evoked response by 40.6 ± 11.1**%** (range 26.5 - 53.0%, *P* = 0.0031, ratio paired *t* test) relative to control (**Figure 5C**). For the same patches, 100 μM Zn^2+^ reduced the GABA-evoked response by 83.9 ± 24.7% (range 41 - 100%, *P* = 0.0423, ratio paired *t* test) relative to control (**Figure 5C**). The block of Zn^2+^ at 10 and 100 µM is consistent with the presence of either a γ subunit or a δ subunit, as well as the absence of the α1 subunit (46).

We next examined the effect of zolpidem, an agonist at the benzodiazepine binding site of the GABA_A_ receptor. Zolpidem has very high affinity for receptors that contain both the α1 and the γ2 subunit (48–50). GABA_A_ receptors with either α2 or α3 subunits (in combination with the γ2 subunit) have reduced sensitivity to zolpidem and receptors with α4, α5 or α6 subunits are essentially insensitive to zolpidem (reviewed in (51)). Thus, zolpidem at a concentration of 100 nM can be used to differentiate receptors with α1 subunits from receptors with either α2 or α3 subunits (e.g., (52)). In addition, zolpidem has virtually no effect at GABA_A_ receptors that contain the γ1 subunit (53) or the γ3 subunit (54). We examined the effect of zolpidem on GABA-evoked responses in nucleated patches, using the same methodology as for Zn^2+^, with repeated application of GABA at 30-s intervals. For the nucleated patch illustrated in **Figures 5D, E**, 100 nM zolpidem had little or no effect on the amplitude or decay time course of the GABA-evoked response. This can be seen from the time series of peak response amplitude (**Figure 5D**) and from the overlay of averaged waveforms for control and 100 nM zolpidem (*n* = 3 successive responses for each condition; **Figure 5E**). For this patch, the average amplitude in control was 45 pA, very similar to the average amplitude of 49 pA in the presence of 100 nM zolpidem.

To examine the potential influence of zolpidem on the response kinetics, we fitted the decay time course of the averaged responses with a triple-exponential function. The weighted decay time constant (τ_w_) was very similar in the control condition (174 ms) and in the presence of zolpidem (164 ms; **Figure 5E**). For a total of four patches, 100 nM zolpidem had no effect on peak amplitude or τ_w_ (**Figure 5F**). In control, the average amplitude was 45.1 ± 9.3 pA (range 32.9 - 55.6 pA) and in 100 nM zolpidem it was 45.0 ± 10.0 pA (range 31.0 - 54.6 pA; *P* = 0.8515, ratio paired *t* test; *n* = 4 patches; **Figure 5F**, top). In control, τ_w_ was 197 ± 65 ms (range 148 - 292 ms) and in 100 nM zolpidem it was 210 ± 61 ms (range 164 - 298 ms; *P* = 0.4584, ratio paired *t* test; *n* = 4 patches; **Figure 5F**, bottom). The lack of effect of zolpidem at 100 nM suggested that the α1 subunit is not present in these GABA_A_ receptors.

We next tested zolpidem at a higher concentration of 1 µM. For the nucleated patch illustrated in **Figures 5G, H**, exposure to 1 µM zolpidem moderately increased the peak amplitude of the GABA-evoked response. This can be seen from the time series of peak response amplitude (**Figure 5G**) and from the overlay of averaged waveforms for control and 1 µM zolpidem (*n* = 3 successive responses for each condition; **Figure 5H**). For this patch, the average amplitude in control was 46 pA and in the presence of 1 µM zolpidem it was 57 pA. When the average response waveforms in control and in 1 µM zolpidem were normalized (by the peak amplitude), the decay kinetics appeared very similar in the two conditions (**Figure 5H**, inset). For quantitative analysis, we fitted the decay with a triple-exponential function and calculated τ_w_. In the control condition, τ_w_ was 327 ms and in 1 µM zolpidem it was 332 ms. For a total of five patches, 1 µM zolpidem resulted in a small, but significant increase of the peak amplitude, but had no effect on τ_w_ (**Figure 5I**). In control, the average amplitude was 30.7 ± 12.1 pA (range 13.1 - 46.1 pA) and in 1 µM zolpidem it was 35.0 ± 15.2 pA (range 14.4 - 57.2 pA; *P* = 0.0270, ratio paired *t* test; *n* = 5 patches; **Figure 5I**, top). In control, τ_w_ was 279 ± 45 ms (range 208 - 327 ms) and in 1 µM zolpidem it was 288 ± 69 ms (range 206 - 377 ms; *P* = 0.9226, ratio paired *t* test; *n* = 5 patches; **Figure 5I**, bottom). In conclusion, the increase of peak amplitude with 1 µM zolpidem suggests the presence of either the α2 or α3 subunit in combination with the γ2 subunit.

### No evidence for δ subunits in GABA_A_ receptors of AII nucleated patches

The sensitivity of the somatic receptors to relatively low concentrations of Zn^2+^ could also suggest the presence of the δ subunit (46). To investigate this, we examined the effect of THIP, a GABA_A_ receptor agonist with high selectivity for receptors containing the δ subunit (55, 56), on nucleated patches. Although THIP is often used at higher concentrations, only responses evoked by ≤1 µM can be unequivocally attributed to the presence of the δ subunit (57–59). For the patch illustrated in **Figure 5J**, application of 1 µM THIP did not evoke a measurable response and had no effect on the membrane noise that could suggest an increase in channel gating. Similar results were observed for a total of four patches tested with 1 µM THIP. In contrast, with 10 µM THIP we observed a clear increase in membrane noise, as well as a small inward current. An example of this can be observed for the patch illustrated in **Figure 5K**. Similar results were observed for a total of three patches tested with 10 µM THIP. The lack of response to 1 µM THIP suggests that the δ subunit is not present in the somatic GABA_A_ receptors of AII amacrine cells.

## Discussion

In this study we performed electrophysiological and pharmacological experiments to investigate GABA_A_ receptors of AII amacrine cells. The AII amacrine cell arguably constitutes the hub of the most dense and complex network of neural connections in the mammalian retina (7) and plays a crucial role in processing both photopic and scotopic signals (6). As such, it is of great interest to characterize the neurotransmitter receptors that mediate synaptic and extrasynaptic inputs to these cells. Surprisingly, we did not observe GABA-mediated IPSCs in these cells, either as spontaneous events or following application of high-K^+^ solution to evoke synaptic release. The frequency of different types of spPSCs observed in retinal neurons can be highly variable (see e.g. refs. 17, 24, and 38 for previous studies of amacrine cells from our laboratory) and we have no explanation for the lack of GABAergic spIPSCs in AII amacrine cells. We speculate that this corresponds to a genuine lack of release under our recording conditions and we consider it very unlikely that the lack of observed GABA-mediated spIPSCs can be explained by technical aspects of the whole-cell recordings (e.g., poor voltage or space clamp, high noise level, etc.). Earlier work from our laboratory demonstrated significant frequency-dependent attenuation in AII cells, most pronounced for signals generated at the arboreal dendrites and propagating towards the cell body (60), but not to the extent that current responses generated by realistic conductance waveforms of spontaneous synaptic input will be completely absent in whole-cell, voltage-clamp recordings.

It is unclear why application of high-K^+^ solution was unable to evoke detectable release from putative GABAergic amacrine cells presynaptic to AII amacrines. From ultrastructural studies, there is clear evidence for synaptic vesicles in amacrine cell processes presynaptic to AIIs (10, 34, 36), and some of these are likely to belong to GABAergic cells. Thus, it seems unnecessary to speculate that putative release of GABA is instead driven by non-vesicular mechanisms. When we applied high-K^+^ solution to AII amacrine cells without blocking glutamatergic synaptic input, we observed a clear increase of PSCs, suggesting increased synaptic release of glutamate from bipolar cells. For A17 amacrine cells, application of high-K^+^ solution (with glutamate and glycine receptors blocked) evoked putative GABAergic PSCs. It is possible that the synaptic release mechanism of GABAergic amacrine cells presynaptic to AII amacrines has a particularly high threshold such that the level of depolarization obtained with high-K^+^ solution is insufficient to trigger release.

We did however, identify GABA_A_ receptor-mediated responses in whole-cell recordings, most likely mediated by receptors located in the dendritic tree, as well as in nucleated patch recordings, mediated by receptors in the soma and the proximal part of the apical dendrite. The GABA-evoked responses in nucleated patches displayed notably slow deactivation kinetics. If the putative synaptic receptors have similar properties, this suggests the capability for pronounced temporal summation. The pharmacology of these receptors ruled out the presence of the α1 subunit and instead suggested they are likely to include α2 and/or α3 subunits, together with the γ2 subunit. Although it is well known from ultrastructural studies that AII amacrine cells receive substantial inputs from other amacrine cells (7, 10), some of which are certainly GABAergic, only some of these inputs have been unequivocally identified with respect to their cellular identity, i.e., dopaminergic amacrine cells (34) and NOS-1 amacrine cells (36). The GABA_A_ receptors of the nucleated patches investigated in this study are located at or close to the AII cell body. Although we cannot know if they are extrasynaptic or synaptic receptors, it is possible that they correspond to synaptic receptors that mediate GABAergic input from dopaminergic amacrine cells which target the soma and apical dendrite of AIIs (34). The extent to which their properties are representative of GABA receptors elsewhere on the AIIs (synaptic and/or extrasynaptic) is not known and must be investigated further.

### Kinetic properties and molecular identity of somatic GABA_A_ receptors of AII amacrine cells

GABA is the major inhibitory neurotransmitter in the adult mammalian CNS (for review, see (61)). Ionotropic GABA receptors of the GABA_A_ subtype are pentamers, composed of two α (α1-α6) subunits and two β (β1-β3) subunits, as well as one accessory subunit (γ1-γ3, δ, ε, π, θ). Different subunit combinations result in receptors with different biophysical properties and different contributions to signal processing in neurons (for review, see (62)). The most common subunit combination of GABA_A_ receptors in the CNS is αβγ, with ~60% of all synaptic receptors composed of α1βγ2 (51). Assemblies with α2 or α3 make up ~35% of GABA_A_ receptors and are common in the hippocampus and striatum (63). The frequent association of γ subunits with synaptic receptors is likely related to their role in anchoring the receptor complex to scaffolding proteins in the postsynaptic density (64). With the exception of α5, all the GABA_A_ receptor subunits have been found to be present in the rodent retina (e.g., (65, 66)).

For the GABA_A_ receptors in AII nucleated patches, the time course of deactivation after a brief pulse was best fitted by a triple-exponential function, similar to what has been observed for GABA-evoked responses in heterologous expression systems (e.g., 52, 67, 68). For the AII responses, the three exponential components of deactivation had well-separated time constants and each made a significant contribution to the response (τ_1_ ~10 ms, 41% amplitude contribution; τ_2_ ~96 ms, 34% amplitude contribution; τ_3_ ~520 ms, 25% amplitude contribution; τ_w_ ~163 ms). Importantly, for some GABA_A_ receptor-mediated spIPSCs, a good fit of the decay phase also requires a triple-exponential function (69).

As is generally the case for ionotropic neurotransmitter receptors, the specific subunit composition of GABA_A_ receptors determines unique functional and kinetic properties (for review, see (70)). For recombinant GABA_A_ receptors, the α subunits play the key role in determining the gating kinetics, with α1-containing receptors exhibiting much faster decay kinetics than receptors containing either α2 or α3 subunits (68, 71–73) (for review, see (62)). Because the experimental conditions can differ in important ways, and because the information required for detailed comparisons is often incomplete, it is not trivial to compare measurements of receptor kinetics between different studies. Nevertheless, we find that our results correspond very well with the deactivation time course of recombinant receptors with an α3β2γ2 subunit composition, reported as τ_1_ = 8.4 ms, τ_2_ = 77.5 ms, τ_3_ = 645 ms and τ_w_ = 185 ms (68). In contrast, the deactivation kinetics for recombinant receptors with an α1β2γ2 subunit composition are approximately three times faster (τ_w_ = 52 ms) (68). The time course of desensitization that we observed with long (1 s) GABA pulses, with τ_fast_ ~60 ms (49% amplitude contribution) and τ_slow_ ~580 ms (τ_w_ ~333 ms), is also remarkably similar to that reported by for the α3β2γ2 composition and much slower than that for the α1β2γ2 composition (68). From another study of recombinant GABA_A_ receptors (72), we calculated a deactivation τ_w_ of ~200 ms for receptors with the α2β1γ2 subunit composition, which is also very similar to our results. In contrast, τ_w_ for receptors with the α1β1γ2 subunit composition is approximately 10 times faster (~20 ms) (72). Taken together, the slow kinetic properties of the GABA_A_ receptors in AII somatic patches suggest that it is unlikely that α1-containing receptors are present to any significant extent.

Similar to our measurements of response kinetics in patches, the studies with which we compared our estimates of kinetic response parameters were also performed at room temperature (68, 72). It is challenging to obtain patch response data at higher, physiologically relevant temperatures, as well as at more than a single temperature when investigating the temperature dependence of ion channels. Importantly, the temperature dependence of receptor kinetics, including synaptic kinetics, tends to be steep, with a *Q*_10_ temperature coefficient (the experimentally determined change for a 10˚C difference in temperature) of 2 - 3. In contrast, the *Q*_10_ of the conductance of an open ion channel is lower (1.2 - 1.5; for detailed discussions in previous studies from our laboratory, see refs. 17, 18, 24, and 38). Whereas *Q*_10_ values for receptor kinetics ideally should be determined experimentally, it is often necessary to scale kinetic data by default values for *Q*_10_, e.g., for purposes of computational modeling.

### Pharmacological properties and molecular identity of the somatic GABA_A_ receptors of AII amacrine cells

In some cases, specific subunits and subunit combinations can be resolved using a pharmacological approach (for review, see (74)). For AII nucleated patches, the reduction of GABA-evoked responses by relatively low concentrations of Zn^2+^ suggested that the subunit composition is either αβγ or αβδ. However, our experiments with the specific δ subunit agonist THIP suggested that this subunit is not present in receptors of AII nucleated patches, consistent with immunolabeling studies which found expression of the δ subunit restricted to cholinergic amacrine cells (65, 75). The ability of relatively low concentrations of Zn^2+^ to suppress GABA-evoked responses of AII nucleated patches also suggested that the α1 subunit is not present (46, 47). This conclusion was supported by the lack of effect of 100 nM zolpidem on the GABA-evoked responses. In contrast, the potentiation by 1 µM zolpidem suggested the presence of the α2 or the α3 subunit (51), as well as the presence of the γ2 subunit (53, 54). These results are consistent with our kinetic analysis which also suggested an αβγ composition with the α2 and/or α3, but not the α1 subunit. Taken together, the results obtained with electrophysiological recording and pharmacology suggest that the GABA_A_ receptors on AII amacrines are predominantly composed of α2 and/or α3 subunits in combination with an (unidentified) β subunit and the γ2 subunit. We did not investigate the presence of β subunits as discrimination of these subunits based on pharmacological experiments is hampered by the lack of specific pharmacological tools (e.g., (76)). Importantly, the β subunit is required for assembly of functional GABA_A_ receptors (77) and all three β subunits are expressed in the mammalian retina (66).

### Functional importance of GABAergic inputs to AII amacrine cells

The AII displays a bistratified morphology, with lobular dendrites that stratify in the OFF-sublamina (*a*) and arboreal dendrites that stratify in the ON-sublamina (*b*) of the inner plexiform layer (10, 78, 79). The inhibitory inputs from other amacrine cells to AIIs are located throughout the dendritic arbors and also close to the soma (7, 10, 11, 34, 36). From serial reconstruction at the ultrastructural level, it has been suggested that AIIs receive synaptic input from at least two different glycinergic and four different GABAergic amacrines (7), but the cellular identity and functional role of the different inputs are unclear.

One potential source of GABAergic input to the vitread side of AII cell bodies is from dopaminergic amacrine cells (27, 32, 34, 80). The processes of dopaminergic amacrine cells appear to form “rings” around the AII somata, but the functionally important relationship is the electron microscopic evidence for pre- and postsynaptic specializations in the dopaminergic and AII amacrine cells (34). Although rodent dopaminergic amacrine cells are thought to release both dopamine and GABA (81, 82) (but see (83) for rabbit retina), only GABA_A_ receptors were found at the synapses made by these cells onto AII amacrine cells (34). Specifically, combined immunolabeling and confocal microscopy suggested the presence of the α1 and α3 subunits. Because dopaminergic amacrine cells themselves express both α1 and α3 subunits (76, 84), it is difficult to unequivocally assign the immunolabeling to the AII using confocal microscopy, but the conclusion with respect to the α3 subunit is consistent with our results.

From other systems, there is evidence that inhibitory inputs targeting different dendritic regions or subcellular compartments of a neuron can serve different and highly specific functions, e.g., input from stellate cells and basket cells that target dendrites and the soma/axon initial segment compartments, respectively, of cerebellar Purkinje cells (for review, see (85)). For AII amacrine cells, the potential functional specificity of inhibitory inputs targeting different cellular compartments is not known. Inhibitory GABAergic input near the soma of AII amacrines has been suggested to shift the balance of AII outputs between the ON- and OFF-pathways (80). The visual receptive field properties of AII amacrines display an ON-center/OFF-surround organization (86–88) thought to be mediated by GABAergic feedback inhibition to rod bipolar cell axon terminals (89). However, it was recently suggested that GABAergic input directly to the AIIs from the NOS-1 amacrine cells may also play an important role in establishing the receptive field surround (36). The very slow kinetics of AII GABA_A_ receptors might be advantageous for maintaining high-fidelity signaling through the large range of light intensities in which the AII network is active (cf. (36)).

In conclusion, if synaptic GABA_A_ receptors of AII amacrines display similarly slow decay kinetics as observed for the receptors of nucleated patches, it could be a functional adaptation for tonic, sustained action, rather than temporal precision (cf. (68)). The slow kinetics will facilitate extensive temporal summation, even at relatively low rates of presynaptic release. This contrasts with the much faster decay kinetics of the glycine receptors of AII cells (24, 25), which are matched to the fast kinetics of the excitatory synaptic input (17). An important next step will be to identify the inhibitory neurons that are presynaptic to AII amacrine cells and to investigate their release properties. Also important will be to determine the functional consequences of activating slow GABA_A_ receptors and fast glycine receptors, how different sources of inhibitory input target specific subcellular regions of AII amacrine cells, and how inhibitory and excitatory inputs are integrated and interact with signals from electrical synapses that mediate homologous (AII - AII) and heterologous (AII - ON-cone bipolar) coupling.

## Data availability statement

The raw data supporting the conclusions of this article will be made available by the authors, without undue reservation.

## Ethics statement

The animal study was reviewed and approved by the Animal Laboratory Facility at the Faculty of Medicine at the University of Bergen (accredited by AAALAC International).

## Author contributions

PB-M and MV performed experiments. PB-M, EH and MV analyzed data and interpreted results. PB-M and MV prepared figures. EH contributed analytic tools. EH and MV conceived and designed the research and wrote the manuscript. All authors contributed to the article and approved the submitted version.
